# Effect of routine preoperative screening for aortic calcifications using noncontrast computed tomography on stroke rate in cardiac surgery: the randomized controlled CRICKET study

**DOI:** 10.1007/s00330-021-08360-4

**Published:** 2021-11-16

**Authors:** Wiebe G. Knol, Judit Simon, Annemarie M. Den Harder, Margreet W. A. Bekker, Willem J. L. Suyker, Linda M. de Heer, Pim A. de Jong, Tim Leiner, Béla Merkely, Miklós Pólos, Gabriel P. Krestin, Eric Boersma, Peter J. Koudstaal, Pál Maurovich-Horvat, Ad J. J. C. Bogers, Ricardo P. J. Budde, on behalf of the CRICKET investigators

**Affiliations:** 1 Department of Cardiothoracic Surgery, Erasmus University Medical Center, Rotterdam, The Netherlands; 2 Department of Radiology and Nuclear Medicine, Erasmus University Medical Center, PO BOX 2040, ND-547, 3000-CA Rotterdam, The Netherlands; 3 Department of Cardiology, Heart and Vascular Center, Semmelweis University, Budapest, Hungary; 4 Department of Radiology, University Medical Center Utrecht and Utrecht University, Utrecht, The Netherlands; 5 Department of Cardiothoracic Surgery, University Medical Center Utrecht, Utrecht, The Netherlands; 6 Department of Cardiovascular Surgery, Heart and Vascular Center, Semmelweis University, Budapest, Hungary; 7 Department of Clinical Epidemiology, Erasmus University Medical Center, Rotterdam, The Netherlands; 8 Department of Neurology, Erasmus University Medical Center, Rotterdam, The Netherlands; 9 Department of Radiology, Medical Imaging Centre, Semmelweis University, Budapest, Hungary

**Keywords:** Tomography, X-ray computed, Cardiac surgical procedures, Stroke, Preoperative care, Radiography

## Abstract

**Objectives:**

To evaluate if routine screening for aortic calcification using unenhanced CT lowers the risk of stroke and alters the surgical approach in patients undergoing general cardiac surgery compared with standard of care (SoC).

**Methods:**

In this prospective, multicenter, randomized controlled trial, adult patients scheduled for cardiac surgery from September 2014 to October 2019 were randomized 1:1 into two groups: SoC alone, including chest radiography, vs. SoC plus preoperative noncontrast CT. The primary endpoint was in-hospital perioperative stroke. Secondary endpoints were preoperative change of the surgical approach, in-hospital mortality, and postoperative delirium. The trial was halted halfway for expected futility, as the conditional power analysis showed a chance < 1% of finding the hypothesized effect.

**Results:**

A total of 862 patients were evaluated (SoC-group: 433 patients (66 ± 11 years; 74.1% male) vs. SoC + CT-group: 429 patients (66 ± 10 years; 69.9% male)). The perioperative stroke rate (SoC + CT: 2.1%, 9/429 vs. SoC: 1.2%, 5/433, *p* = 0.27) and rate of changed surgical approach (SoC + CT: 4.0% (17/429) vs. SoC: 2.8% (12/433, *p* = 0.35) did not differ between groups. In-hospital mortality and postoperative delirium were comparable between groups. In the SoC + CT group, aortic calcification was observed on CT in the ascending aorta in 28% (108/380) and in the aortic arch in 70% (265/379).

**Conclusions:**

Preoperative noncontrast CT in cardiac surgery candidates did not influence the surgical approach nor the incidence of perioperative stroke compared with standard of care. Aortic calcification is a frequent finding on the CT scan in these patients but results in major surgical alterations to prevent stroke in only few patients.

**Key Points:**

• *Aortic calcification is a frequent finding on noncontrast computed tomography prior to cardiac surgery.*

• *Routine use of noncontrast computed tomography does not often lead to a change of the surgical approach, when compared to standard of care.*

• *No effect was observed on perioperative stroke after cardiac surgery when using routine noncontrast computed tomography screening on top of standard of care.*

**Supplementary Information:**

The online version contains supplementary material available at 10.1007/s00330-021-08360-4.

## Introduction

Stroke is a feared complication in general cardiac surgery, occurring on average in 2% of patients [1]. The majority of perioperative strokes is caused by embolization for which aortic atherosclerosis is a major risk factor [2]. In most cardiac surgical procedures, the surgeon manipulates the aorta, for instance when introducing aortic cannulas, clamping the aorta, creating proximal anastomoses, or incising the aorta to gain access to the aortic valve. Each manipulation can dislodge atherosclerotic debris causing embolic stroke.

Intraoperatively, the presence of atherosclerosis in the ascending aorta can be examined by means of manual palpation, epiaortic ultrasound, or transesophageal echocardiography [3]. Using epiaortic ultrasound to guide surgical approach lowers the risk of stroke [4]. However, because these techniques can only be applied intraoperatively, the options to alter the approach are limited and decisions need to be taken ad hoc. Preoperative imaging has been used to detect aortic calcifications, including routinely performed conventional chest radiograph (CXR). However, CXR findings do not correlate strongly with the risk of stroke [5]. The European Society of Cardiology and European Association for Cardio-Thoracic Surgery guideline on myocardial revascularization mention noncontrast CT screening as an option in patients with a high risk of stroke [6]. Several non-randomized studies indeed found a reduced stroke and mortality rate when using preoperative CT imaging [7], but randomized studies are lacking. Therefore, we initiated a parallel group randomized controlled trial, the “ultra low-dose chest CT with iterative reconstructions as an alternative to conventional CXR prior to heart surgery” (CRICKET) study, of which the design has previously been published [8]. The principal aim of the study was to evaluate if routine use of adding a preoperative unenhanced chest CT scan to standard of care lowers the risk of perioperative stroke and alters the surgical approach in patients undergoing general cardiac surgery, when compared with standard of care (SoC) alone, which includes a CXR, and a CT scan only when deemed clinically indicated by the physician.

## Materials and methods

Three centers (listed in the Supplementary data) enrolled patients for the study. This prospective randomized controlled study was approved by the medical ethical committee (13–692/M) and local approval was obtained in all participating centers. The study adhered to the declaration of Helsinki and patients provided written informed consent. The study was registered at www.clinicaltrials.gov (NCT02173470) and the Dutch Trial Register (NL4336). The study protocol has previously been published [8].

### Study sample

From September 2014 to October 2019, patients (≥ 18 years old) scheduled to undergo elective cardiac surgery in one of the participating centers were screened for eligibility. Cardiac surgery was defined as all surgical procedures on the structural heart through median sternotomy or thoracotomy. Exclusion criteria were pregnancy, emergency surgery, when a chest or cardiac CT had been performed in the past 3 months, concomitant or previous participation in a study that exposed the patient to radiation, and unwillingness to be informed about incidental findings on the CT images.

### Randomization

Eligible patients were included by the investigators, either during their preoperative visit to the outpatient clinic or during admission the day before surgery. Patients were randomized 1:1 to the intervention (SoC + CT group) or to receive standard of care (SoC group). A web-based randomization module was used, ensuring concealment of allocation to the investigators until group assignment. The module used blocks of eight and stratification of participating centers.

### Intervention

The SoC group received a standard of care, which included a CXR. The CXR was reported as part of the normal clinical workup, with special emphasis on the presence of aortic calcification. The presence of calcification was scored for the ascending aorta specifically and for any presence of calcification in the ascending aorta or the aortic arch at all. In all regards, the management of the SoC group was according to local clinical practice and was not influenced by inclusion in the study. The standard of care group did not undergo a CT scan as part of any targeted screening approach. Any preoperative CT scan that was performed in this group was at the discretion of the physician. The number and reason for these scans were reported. Patients in the SoC + CT group received standard of care and additionally a noncontrast chest CT. The CT scan was acquired either during the pre-operative outpatient visit or the day before surgery. The scan range was set to include at least the proximal aortic arch branches cranially and the entire heart caudally. Only CT scanners with ≥ 64 detectors were used. Tube voltage and current were chosen at the discretion of the local hospitals, aiming for an effective radiation dose below 1 milliSievert (mSv). No ECG-gating was used. As voltages ranged from 80 to 130 kV, effective radiation dose was calculated, based on the dose-length product, using a conversion factor of 0.0147 for all scans [9]. Images were reconstructed with a slice thickness of ≤ 1 mm and a section interval of ≤ 1 mm in the axial plane, with the possibility of multiplanar reformatting. The CT scan was reported by a radiologist using a standardized reporting template, including evaluation of the ascending aorta for any calcification on the ventral side, calcifications > 1 cm in diameter, or calcifications spanning at least half the circumference. The ventral side was chosen because this side is most frequently manipulated during surgery. The presence of any calcification in the aortic arch, defined as the part between the origin of the brachiocephalic artery to the origin of the left subclavian artery, was scored after data collection. The surgeon decided whether the surgical approach should be altered or not. This decision was recorded on a separate standardized form for each patient and included the surgeon’s consideration. Preoperative Doppler evaluation of the carotid arteries and intraoperative epi-aortic ultrasound to screen for atherosclerosis were not routinely used in any of the participating centers. After the study was completed, an Agatston calcium score was calculated for the ascending aorta and aortic arch with the same boundaries, using a standard clinical Agatston Calcium Scoring tool (Intellispace Portal, Philips Healthcare) [10]. The threshold was not adjusted to tube voltage, accepting potential overestimation of the Agatston score in scans with lower tube voltages.

### Endpoints

All endpoints were assessed until discharge from the hospital. The primary endpoint of the study was perioperative stroke. Stroke was defined as the presence of acute focal neurological signs or symptoms, with corresponding infarction on cerebral CT-scan or MRI scan, or absence of other apparent causes. Cerebral imaging was performed at the clinicians’ discretion. Additional information on the primary endpoint is summarized in the Supplementary data. The secondary endpoints were change in surgical approach, in-hospital mortality, and postoperative delirium. According to the initial protocol, the change in approach was to be described only in the SoC + CT group. However, at the time of data completion, it was decided to compare the rate of change in approach between the two study arms. A change in approach was defined as any change in the surgical plan between the inclusion of the patient and the start of the operation, including postponement or cancellation of surgery. Cases in which the patient refused surgery independently and on their own initiative were not considered a change of approach. Intraoperative changes were not included. Because stroke is associated with delirium and mortality, the latter two were added as secondary endpoints. Additional information on the analysis of the endpoints is described in the supplementary methods (Supplementary data). Endpoints were evaluated with an intention-to-treat analysis.

### Sample size calculation

As was previously described, the sample size was calculated using the local incidence of stroke at the initiating center [8]. A 2.0% rate of stroke was assumed for the SoC group. From previous literature, it was estimated that the reduction of stroke in the SoC + CT group would be fourfold; thus, a stroke rate of 0.5% was assumed [11]. A two-tailed test with an *alpha*-level of 0.05 and a statistical power of 0.80 was used, resulting in a sample size of 1724 patients in total.

### Interim-analysis

Because of a slower than expected inclusion rate it was decided during the study to do an interim analysis after the inclusion of half the desired sample size. After consultation of a statistician (E.B.), we calculated that the chance of observing the hypothesized stroke reduction under the current trend was < 1%. As a result, it was decided to halt the study. Additional information on the interim analysis is provided in the Supplementary data.

### Statistical analysis

Continuous variables were described as mean ± standard deviation in case of a normal distribution, or as a median with interquartiles otherwise. Categorical variables were described as frequencies and percentages. Radiation dose and hospital stay were compared using the Mann–Whitney U test. Differences between the two groups in primary and secondary endpoints were evaluated using Fisher’s exact test, using the Clopper-Pearson exact method to calculate 95% confidence intervals. Statistical analyses were performed using SPSS software version 25 (SPSS Inc). A *p* value of 0.05 was used to determine statistical significance.

## Results

### Study sample characteristics

**Fig. 1:**
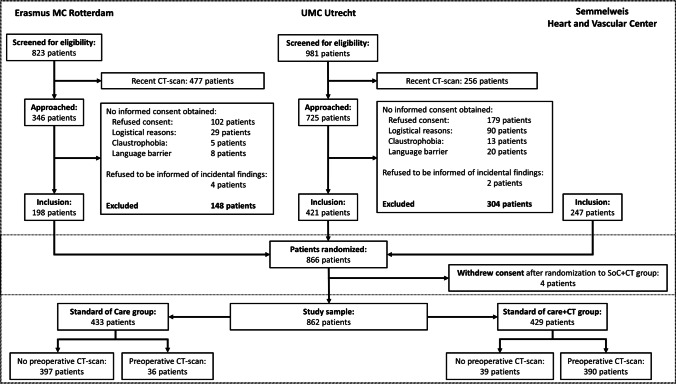
Study flowchart. Screening data was available in two of the three participating centers

A total of 866 patients were randomized from the start of the study until the interim analysis, as is shown in the flowchart (Fig. 1). Four patients, all randomized to the SoC + CT group, withdrew consent to use their data prior to the scan, leaving 433 patients in the SoC group (mean age, 66 years ± 11; 74.1% male) and 429 patients in the SoC + CT group (mean age, 66 years ± 10; 69.9% male) available for analysis. Baseline characteristics of these patients, including the type of planned surgery, are shown in Table 1. The distribution of types of planned surgery is given in Table 2.

**Table 1:** Baseline characteristics

Characteristic	SoC group (*n* = 433)	SoC + CT group (*n* = 429)
Age (years, mean ± SD; range)	66 ± 11; (23—88)	66 ± 10; (26 – 98)
Sex		
*Male*	74.1% (321/433)	69.9% (300/429)
*Female*	25.9% (112/433)	30.1% (129/429)
Diabetes		
*Oral medication*	15.5% (67)	11.9% (51)
*Insulin dependent*	7.6% (33/433)	6.8% (29/429)
Hypertension	65.3% (282/432)	68.3% (291/426)
Smoking		
*Currently*	12.3% (52/424)	13.7% (57/417)
*Stopped smoking*	41.7% (177/424)	37.6% (157/417)
COPD	10.6% (46/433)	13.5% (58/429)
Chronic kidney disease	15.9% (69/433)	17.0% (73/429)
Dialysis	0.9% (4/433)	0.2% (1/429)
Peripheral obstructive arterial disease	6.7% (29/433)	5.6% (24/429)
Prior cerebrovascular accident	5.8% (25/433)	5.6% (24/429)
Prior transient ischemic attack	5.1% (22/433)	5.8% (25/429)
Atrial fibrillation	16.4% (71/433)	17.8% (76/429)
EuroScore II (in %, median [Q1 –Q3])	1.31% [0.85–2.29%]	1.35% [0.88–2.27%]
Prior myocardial infarction	19.0% (82/432)	16.3% (70/429)
Recent myocardial infarction	3.7% (16/430)	4.2% (18/428)
Reoperation	1.6% (7/432)	2.8% (12/428)
NYHA classification		
*Class I*	20.0% (82/410)	19.9% (82/413)
*Class II*	54.1% (222/410)	50.8% (210/413)
*Class III*	21.0% (86/410)	26.6% (110/413)
*Class IV*	4.9% (20/410)	2.7% (11/413)

No significant differences were present between groups after randomization. Proportions are given as % (*n*)

*COPD* chronic obstructive pulmonary disease, *NYHA* New York Heart Association

**Table 2:** Type of surgery planned

Type of surgery	SoC group	SoC + CT group
Isolated CABG	39.5% (171/433)	37.1% (159/429)
Isolated AVR^a^	21.0% (91/433)	25.4% (109/429)
AVR + CABG	11.3% (49/433)	8.6% (37/429)
Mitral valve surgery^a^	17.6% (76/433)	20.7% (89/429)
Other	10.6% (46/433)	7.9% (34/429)

No significant differences were present between groups after randomization. Proportions are given as % (*n*). A full list of other types of surgery is provided in the Supplementary Data

^a^A minimally invasive surgical approach was used in four patients: mitral valve surgery (2 patients), septal defect closure (1 patient), and aortic valve replacement (1 patient)

*AVR* aortic valve replacement, *CABG* coronary artery bypass grafting

### Preoperative CXR and CT scan

In the SoC group, 36 patients (8.3%, 36/433) underwent a CT scan before the operation because of suspected aortic calcification (*n* = 13), suspected pulmonary nodules (*n* = 5), suspected dilatation of the aorta (*n* = 5), prior to minimally invasive surgery (*n* = 3), due to a protocol violation (*n* = 3), for other suspected masses on CXR (*n* = 2), for other pulmonary abnormalities on CXR (*n* = 2), or other reasons (*n* = 3). The scan protocols of these scans varied based on the clinical indication. In the SoC + CT group, 39 patients (9.1%, 39/429) did not undergo the study CT scan, because of logistic reasons (*n* = 30), the patient ultimately refused the scan (*n* = 6), surgery was canceled prior to the scan (*n* = 1), or an unknown reason (*n* = 2).

The prevalence of aortic calcification and additional findings on CXR and CT scan are shown in Table 3. Ascending aortic calcification was seen on CXR in 12.6% (54/429) in the SoC + CT group vs. 13.0% (56/432) in the SoC group. The study CT scan in the SoC + CT group showed the presence of aortic calcification in the aortic arch in 69.9% (265/379). In the ascending aorta, calcifications were present in 28.4% (108/380), calcifications at the ventral side in 17.2% (67/389), calcifications ≥ 1 cm in diameter in 11.6% (45/389), and calcification spreading at least half the circumference in 10.0% (39/389). The median radiation dose of the CT scans in the SoC + CT group was 0.68 (1^st^ and 3^rd^ interquartile 0.51, 0.80) mSv, which was lower than the dose of the CT scans performed in the SoC group, with a mean radiation dose of 1.68 (1^st^ and 3^rd^ interquartile 0.79–5.62) mSv (*p* < 0.001) for 28 of 36 scans with available radiation dose reports.

**Table 3:** Results of CXR and CT scan

Imaging characteristic	SoC group	SoC + CT group
Any aortic calcification on CXR	53.2% (230/432)	53.4% (229/429)
Ascending aortic calcification on CXR	13.0% (56/432)	12.6% (54/429)
CT radiation dose (mSv), median [Q1 – Q3]	1.68 [0.79–5.62] (*n* = 28)	0.68 [0.51–0.80]
Percentage of CT scans < 1 mSv	28.6% (8/28)	83.1% (324/390)
Any calcification at ascending aorta on CT	51.4% (18/35)	28.4% (108/380)
Agatston score at ascending aorta (out of participants with calcification), median [Q1 – Q3]	638 [66 – 2225] (*n* = 13)*	293 [92 – 842] (*n* = 103)*
Calcification > 1 cm at ascending aorta on CT	25.7% (9/35)	11.6% (45/389)
Ventral calcification at ascending aorta on CT	14.3% (5/35)	17.2% (67/389)
Calcification at ascending aorta at least half the circumference on CT	5.7% (2/35)	10.0% (39/389)
Any calcification in the aortic arch on CT	94.1% (32/34)	69.9% (265/379)
Agatston score at the aortic arch (out of participants with calcification), median [Q1 – Q3]	1298 [627 – 3067] (*n* = 24)^a^	852 [289 – 2119] (*n* = 260)^a^

Proportions are given as % (*n*)

*CXR* chest radiograph, *SoC* standard of care

^a^An Agatston-score could not be calculated for 8 participants in the SoC group and 6 participants in the SoC + CT group

### Endpoints

**Fig. 2:**
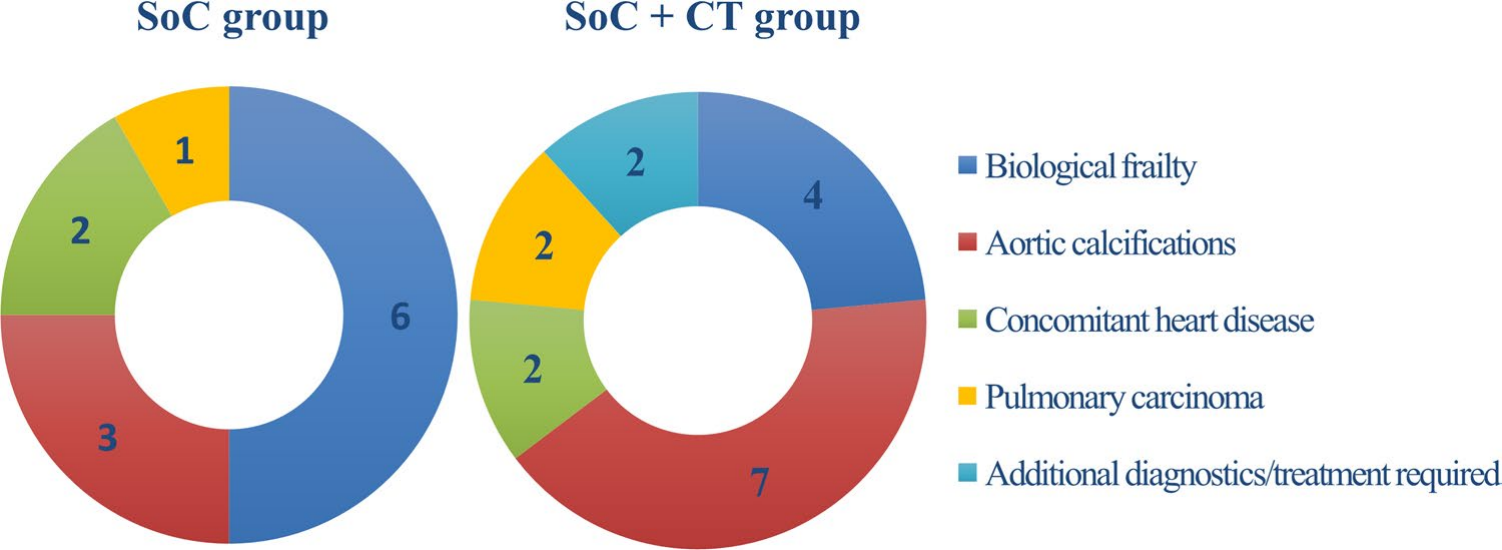
Reasons for changing the surgical approach in both groups

**Fig. 3:**
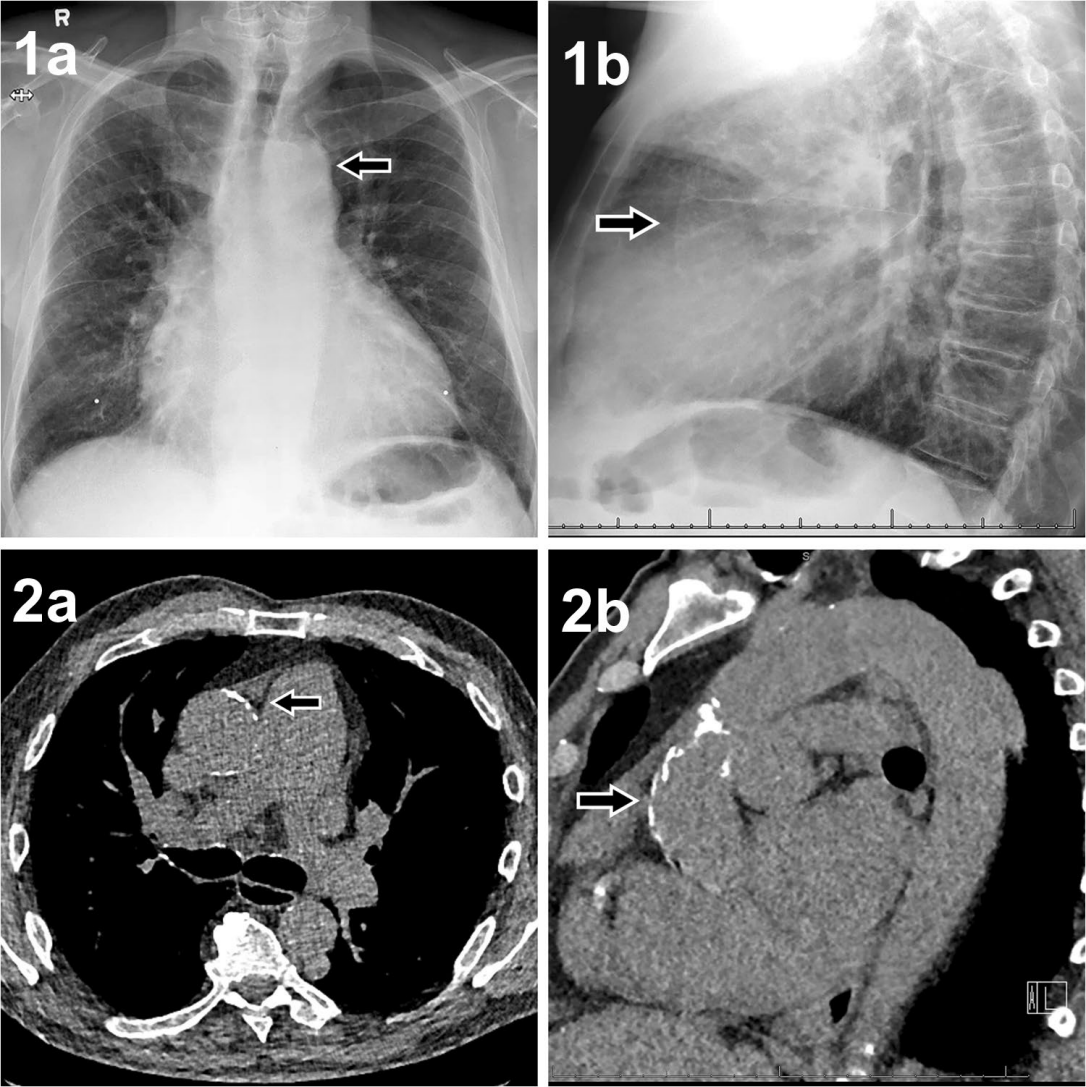
Sample images of CXR and noncontrast CT. Sample images of a 70-year-old male patient whose surgical approach was changed from surgical to transcatheter aortic valve replacement. 1: The posterior-anterior (**1a**) and lateral (**1b**) views of the preoperative CXR 2: Ascending aortic calcifications on the axial (**2a**) and sagittal (**2b**) plane of the noncontrast CT. The arrow in panel **1a** indicates the aortic knob, where only modest calcification is seen. The arrow in panel **1b** point at the ventral boundary of the aorta, where no clear calcifications seem to be present. The arrows in panels **2a** and **2b** indicate the extensive ventral calcifications, hampering aortic manipulation in this area

As is shown in Table 4, the stroke rate was not different between the SoC + CT group and the SoC group (2.1% [1.0–3.9%] (9/429) vs. 1.2% [0.4–2.7%] (5/433), resp., *p* = 0.27). There was no difference between the participating centers (Erasmus MC: 2.0% (4/198), UMC Utrecht: 1.4% (6/421) and Semmelweis Heart and Vascular Center: 1.7% (4/243), *p* = 0.83). The characteristics of the patients who suffered a perioperative stroke are shown in Table S2 and S3 (supplementary data). The surgical approach was changed in 4.0% [2.3–6.3%] (17/429) of the patients in the SoC + CT group vs. 2.8% [1.4–4.8%] (12/433) of the patients in the SoC group (*p* = 0.35). In both groups, the most frequent change of approach was to change surgery to percutaneous treatment (11 patients in the SoC + CT group and 5 patients in the SoC group). Surgery was canceled in one patient in the SoC + CT group (due to new-onset ascites and a reduction in renal and liver function) and three patients in the SoC group (two patients because of too high risk and one after diagnosing a pulmonary malignancy). All reasons to change the approach have been summarized in Fig. 2. In patients with a changed approach, no perioperative stroke was observed. In-hospital mortality (1.4% [0.5–3.0%] (6/429) vs. 0.9% [0.3–2.4%] (4/433), *p* = 0.55) and postoperative delirium rate (7.0% [4.8–9.8%] (30/429) vs. 7.2% [4.9–10.0%] (31/433), *p* = 0.92) were not different between the SoC + CT and SoC group. Sample images of a patient with a change in approach due to aortic calcifications are shown in Fig. 3.

**Table 4:** Study endpoints

Endpoint	SoC group (*n* = 433)	SoC + CT group (*n* = 429)	*p* value
Perioperative stroke	1.2% [0.4 – 2.7%] (5/433)	2.1% [1.0 – 3.9%] (9/429)	0.27
Change of surgical approach	2.8% [1.4 – 4.8%] (12/433)	4.0% [2.3 – 6.3%] (17/429)	0.35
Change to off-pump surgery	2	1	
Additional concomitant surgery	2	2	
Change to percutaneous approach (TAVR or PCI)	5	11	
Postponement of surgery	0	2	
Cancellation of surgery	3	1	
Delirium	7.2% [4.9 – 10.0%] (31/433)	7.0% [4.8 – 9.8%] (30/429)	0.92
In-hospital mortality	0.9% [0.3 – 2.4%] (4/433)	1.4% [0.5 – 3.0%] (6/429)	0.55
Hospital stay (days, median [Q1-Q3])	6 [4–7]	6 [4–7]	0.53

Endpoints in both study arms, based on an intention to treat principle. Proportions are given as % [95% confidence interval] (*n*)

*PCI* percutaneous coronary intervention, *SoC* standard of care, *TAVR* transcatheter aortic valve replacement

## Discussion

In this multicenter randomized controlled trial, we found that routine preoperative noncontrast-enhanced chest CT screening for aortic calcifications in addition to standard of care, including CXR, did not lead to a reduced perioperative stroke rate. Adding noncontrast CT did not increase the rate of change in the surgical approach, when compared with SoC alone. The perioperative stroke rate did not differ between groups. In the patients in the SoC + CT group, aortic calcification was present in the ascending aorta in 28% and in the aortic arch in 70%.

A review of non-randomized studies evaluating the effect of preoperative CT screening in primary cardiac surgery found a reduction in both the rate of stroke and mortality [7]. In the included studies, the surgical approach was changed in up to 13–17% of patients [11, 12]. In our study, however, the change of approach was only 4.0%. Without a changed approach, the preoperative CT scan cannot prevent perioperative stroke and will not influence this risk. We propose three factors underlying this low rate of change.

First, we used an all-comers design, enrolling all adult patients undergoing general cardiac surgery, rather than targeting a specific population based on the risk of aortic calcification [11, 12]. Applying such high-risk features as inclusion criteria to our study population would identify approximately half of the patients as candidates for targeted screening [11]. Only one previous study enrolled patients undergoing general cardiac surgery without selection of high-risk patients and they found a comparable changed approach rate of 4.3% [13]. Focused screening will result in a higher prevalence of problematic aortic atherosclerosis, more frequent changes in the approach, and, possibly, a greater reduction in perioperative stroke.

The second reason for the low rate of changed approach could be that the presence of aortic calcification is too non-specific to predict perioperative stroke. A recent propensity-matched retrospective study found that a preoperative contrast-enhanced chest CT scan, allowing visualization of aortic non-calcified plaque, reduced the rate of stroke in patients scheduled for coronary artery bypass grafting [14]. If the association between aortic non-calcified plaque and perioperative stroke is stronger than that of calcified plaque, then a contrast-enhanced CT scan could be a more valuable screening tool.

Finally, the difficulty in estimating the stroke risk based on the degree of aortic calcification poses a dilemma: altering the surgical approach in all patients with clinically significant calcifications would lead to altered surgical approaches in many patients. This scenario is desirable only if the alternative approach results in comparable outcomes with regard to early and long-term survival and quality of life. We observed that this tradeoff was often the reason not to change the approach. Despite the presence of calcifications, the estimated risk of stroke was not high enough to justify changing to the alternative approach.

Although lowering the risk of stroke was the main aim of preoperative CT screening in our study, several other benefits could be expected from this screening. The sensitivity of CT scans to detect aortic calcification is much higher than that of CXR [15]. Although the prevalence of a porcelain aorta is low, screening with CT could detect patients with a porcelain aorta missed on CXR [16]. Also, CT scans can provide the surgeon with other valuable information, such as anatomical abnormalities or concomitant diseases. Our study has shown that preoperative CT screening can be achieved at a radiation dose below 1 mSv in 83% (324/390) of the patients.

Our study has limitations. First, the trial was halted halfway due to expected futility. This reduced the power due to a smaller sample size, increasing the chance of a type 2 error. The interim analysis showed the chance of observing the hypothesized stroke reduction of 75% is < 1%, but a smaller reduction cannot be ruled out. Second, many patients were excluded because they had already undergone a recent CT scan. Thus, certain types of surgery, such as minimally invasive surgery, or patients with evident aortic calcification on other imaging modalities, are not represented in our study sample. Finally, we did not perform routine postoperative neurological examination or cerebral imaging. However, since the effect of screening on perioperative stroke can never exceed the effect that screening has on the change of approach, it is unlikely that the absence of systematical neurological screening biased our results.

Our findings have several implications for future research. More insights into the balance between the risk of stroke and the long-term outcomes of alternative approaches might aid the consideration of whether or not to change the surgical approach. The value of adding contrast to preoperative CT should be explored further, as it enables direct imaging of atheromatous plaque. This potentially increases the diagnostic value of CT to predict the risk of stroke. Finally, the benefits of additional information on porcelain aorta, patient anatomy and concomitant diseases provided by preoperative CT screening deserve further evaluation, weighing them against the downsides of radiation dose, health care costs, and the burden of incidental findings.

In summary, the routine use of a preoperative non-contrast enhanced chest CT scan to screen for aortic calcification in all patients scheduled for cardiac surgery did not lead to a change in surgical approach more often than the standard of care. Aortic calcification was a frequent finding on the CT scan in these patients, but resulted in major surgical alterations to prevent stroke in only few patients. Future research is needed to evaluate the use of contrast enhancement and patient selection to improve the efficiency of preoperative screening.

## Supplementary Information

Below is the link to the electronic supplementary material.

Supplementary file1 (DOCX 31 KB)

## Abbreviations

AVR: Aortic valve replacement
CABG: Coronary artery bypass grafting
COPD: Chronic obstructive pulmonary disease
CXR: Chest radiograph
NYHA: New York Heart Association
PCI: Percutaneous coronary intervention
SoC: Standard of care
TAVR: Transcatheter aortic valve replacement

## Declarations

### Guarantor

The scientific guarantor of this publication is Ricardo P.J. Budde.

### Statistics and biometry

One of the authors (E.B.) has significant statistical expertise.

### Methodology

• prospective

• randomised controlled trial

• multicenter study
