# Supplementary material for: Effect of routine preoperative screening for aortic calcifications using noncontrast computed tomography on stroke rate in cardiac surgery: the randomized controlled CRICKET study
Source: Eur Radiol. 2021 Nov 16;32(4):2611–9. doi: 10.1007/s00330-021-08360-4 (PMC8921026; doi:10.1007/s00330-021-08360-4)
Supplement: Supplementary file 1 — Supplementary file1 (DOCX 31 KB) [file 330_2021_8360_MOESM1_ESM.docx]

**Supplementary data**

**Supplementary data to:** Effect of routine preoperative screening for aortic calcifications using noncontrast computed tomography on stroke rate in cardiac surgery: the randomized controlled CRICKET-study; W.G. Knol, J. Simon, A.M. den Harder et al

Contents

[CRICKET investigators 2](#_Toc65512398)

[University Medical Center Utrecht, the Netherlands: 2](#_Toc65512399)

[Semmelweis Heart and Vascular Center Budapest, Hungary 2](#_Toc65512400)

[Erasmus Medical Center Rotterdam, the Netherlands 2](#_Toc65512401)

[Supplementary methods 4](#_Toc65512402)

[Interim analysis 5](#_Toc65512403)

[Table S1: Type of surgery planned 6](#_Toc65512404)

[Table S2: Primary endpoint evaluation. 7](#_Toc65512405)

[Table S2: Baseline characteristics of participants that suffered a stroke. 8](#_Toc65512406)

[References 9](#_Toc65512407)

# CRICKET investigators

### University Medical Center Utrecht, the Netherlands:

Department of Radiology:

- R.P.J. Budde, MD, PhD
- A.M. Den Harder, MD, PhD
- Prof. P.A. de Jong, MD, PhD
- Prof. Tim Leiner, MD, PhD

Department of Cardiothoracic surgery:

- L.M. de Heer , MD, PhD
- R.C.A. Meijer, MD
- Prof. dr. Willem J.L. Suyker, MD, PhD

Department of Public Health:

- Dr. G.A. de Wit, PhD

### Semmelweis Heart and Vascular Center Budapest, Hungary

Department of Radiology:

- J. Simon, MD
- Prof. P. Maurovich-Horvat, MD, PhD

Department of Cardiology:

- Prof. B. Merkely, MD, PhD

Department of Cardiovascular surgery:

- K. Benke, MD, PhD
- M. Pólos, MD, PhD

### Erasmus Medical Center Rotterdam, the Netherlands

Department of Radiology and Nuclear medicine:

- R.P.J. Budde, MD, PhD
- Prof. G.P. Krestin, MD, PhD

Department of Cardiothoracic surgery:

- W.G. Knol, MD
- M.W.A. Bekker, MD
- Prof. A.J.J.C. Bogers, MD, PhD

Department of Clinical epidemiology:

- Prof. E. Boersma, PhD

Department of Neurology:

- Prof. P.J. Koudstaal, MD, PhD

Supplementary methods

**Endpoints:**

The primary endpoint of the study was perioperative stroke. Stroke was defined as the presence of acute focal neurological signs or symptoms, with corresponding infarction on cerebral CT-scan or MRI scan or absence of other apparent causes. These symptoms could be present either <24 hours or longer, making no distinction between stroke and transient ischemic attack. These definitions are in concordance with a combination of the NeuroARC definitions of type 1: overt CNS injury or type 3.a: transient ischemic attack^1^. Type 2 (covert stroke) could not be evaluated, as participants did not undergo routine MRI of the brain. The decision to perform cerebral imaging was at the discretion of the clinician. Neurologists were consulted in case of clinical suspicion of stroke, as part of the usual clinical care. Of all participants with a suspected stroke, a summary of clinical and imaging information was evaluated by a vascular neurologist (blinded for review), blinded to participant allocation, who finally decided on whether a stroke had likely occurred or not.

Postoperative delirium was defined as any case of delirium that was treated with antipsychotic agents, excluding preventive treatment regimens.

**Statistical analysis:**

Since the aim was to compare routine use of CT with SoC, intention-to-treat analyses were performed for the primary and secondary endpoints. Patients that withdrew consent to use their data prior to the study scan were not included in the analysis. Crossover between groups and the reason why were registered.

# Interim analysis

**Rationale for the interim analysis**

Because of a slower than expected inclusion rate, the decision was made, well before reaching the first half of the sample size, to temporarily halt the inclusion halfway. At this time, all data was combined and a statistician (E.B.) was consulted to evaluate the conditional power to find the hypothesized effect, given the preliminary results.

**Results of the interim analysis**

The conditional power was calculated for the binomial primary study endpoint stroke, using the method described by Proschan et al.^2^ In short, this method can be used to project a test statistic at the end of the study, based on the observed data and an assumption on the underlying effect. Using the values specified in the study protocol (an alpha of 0.05, a beta of 0.20, a two-tailed test, 1:1 randomization, a stroke rate of 2% in the SoC group and 0.5% in the SoC+CT group), the conditional power to find the hypothesized effect under the current trend was <1%.

Subsequently, based on this calculation the authors decided to halt the study for expected futility.

# **Table S1**: Type of surgery planned

| Type of surgery | SoC group | SoC+CT group |
| --- | --- | --- |
| Isolated CABG | 39.5% (171/433) | 37.1% (159/429) |
| Isolated AVR^a^ | 21.0% (91/433) | 25.4% (109/429) |
| AVR+CABG | 11.3% (49/433) | 8.6% (37/429) |
| Mitral valve surgery^a^ | 17.6% (76/433) | 20.7% (89/429) |
| Other | 10.6% (46/433) | 7.9% (34/429) |
| AVR + additional procedure (aneurysmectomy, ablation surgery, tricuspid valve surgery) | 2.3% (10/433) | 3.0% (13/429) |
| CABG + additional procedure (aneurysmectomy, ablation surgery, septal defect) | 2.1% (9/433) | 1.2% (5/429) |
| Aortic (root) surgery | 1.6% (7/433) | 1.2% (5/429) |
| Tricuspid valve surgery | 0.9% (4/433) | 0.5% (2/429) |
| Septal defect* | 1.2% (5/433) | 0.2% (1/429) |
| LVAD implantation | 0.2% (1/433) | 0.7% (3/429) |
| Myectomy (Morrow) | 0.9% (4/433) | 0.5% (2/429) |
| Pulmonary valve surgery | 0.5% (2/433) | 0.7% (3/429) |
| Left ventricular aneurysmectomy | 0.5% (2/433) | 0 |
| Excision of myxoma | 0.5% (2/433) | 0 |
| Closure of coronary fistula | 0 | 0.2% (1/429) |

No significant differences were present between groups after randomization. Proportions are given as % (n).

a) a minimally invasive surgical approach was used in two patients undergoing mitral valve surgery, a patient undergoing closure of a septal defect and a patient undergoing aortic valve replacement.

AVR = Aortic valve replacement, CABG = Coronary artery bypass grafting, LVAD = Left ventricular assist device.

Table S2: Primary endpoint evaluation.

|  | No. of patients |
| --- | --- |
| Participants evaluated for possible perioperative stroke | 20 |
| Suspected of Stroke | 11 |
| Suspected of TIA | 3 |
| Uncertain | 6 |
|  |  |
| Perioperative stroke scored after neurologist evaluation | 14 |
| Stroke | 11 |
| TIA | 3 |
|  |  |
|  |  |
| Characteristics of participants with perioperative stroke: |  |
| Stroke: | 11 |
| Right hemisphere | 6 |
| Left hemisphere | 5 |
| Abnormalities on neuro imaging | 10 |
| TIA: | 3 |
| Right hemisphere | 0 |
| Left hemisphere | 3 |
| Abnormalities on neuro imaging | 0 |

Table S2: Baseline characteristics of participants that suffered a stroke. Proportions are given as % (n). Due to the absolute number of strokes, no statistical inference or comparison with non-stroke participants was performed.

|  |  | Stroke (n=14) |
| --- | --- | --- |
| Age (years ± SD) |  | 68 ± 10 |
| Sex | Male | 71% (10) |
| Diabetes | Oral medication | 29% (4) |
|  | Insulin dependent | 14% (2) |
| Hypertension |  | 86% (12) |
| Smoking | Currently | 21% (3) |
|  | Stopped smoking | 29% (4) |
| Chronic Kidney Disease |  | 21% (3) |
| Peripheral Obstructive Arterial Disease |  | 21% (3) |
| Prior CVA |  | 14% (2) |
| Prior TIA |  | 7% (1) |
| Atrial fibrillation |  | 14% (2) |
| EuroScore II | median [Q1 – Q3] | 1.92% [1.19-4.39%] |
| Reoperation |  | 0 |
| Type of surgery |  |  |
|  | Isolated AVR | 14% (2) |
|  | Isolated CABG | 36% (5) |
|  | AVR+CABG | 29% (4) |
|  | Mitral surgery + CABG | 7% (1) |
|  | Tricuspid valve surgery | 14% (2) |
| CXR |  |  |
|  | Any aortic calcification | 79% (11) |
|  | Ascending aortic calcification | 14% (2) |
| CT |  |  |
|  | Any calcification at ascending aorta | 36% (4/11) |
|  | Any calcification in the aortic arch | 91% (10/11) |

Abbreviations: AVR= Aortic Valve Replacement, CABG = Coronary Artery Bypass Grafting, CVA= Cerebrovascular Accident, TIA= Transient Ischemic Attack,

# References

1. Lansky AJ, Messe SR, Brickman AM, Dwyer M, Bart van der Worp H, Lazar RM, Pietras CG, Abrams KJ, McFadden E, Petersen NH, Browndyke J, Prendergast B, Ng VG, Cutlip DE, Kapadia S, Krucoff MW, Linke A, Scala Moy C, Schofer J, van Es GA, Virmani R, Popma J, Parides MK, Kodali S, Bilello M, Zivadinov R, Akar J, Furie KL, Gress D, Voros S, Moses J, Greer D, Forrest JK, Holmes D, Kappetein AP, Mack M, Baumbach A. Proposed Standardized Neurological Endpoints for Cardiovascular Clinical Trials: An Academic Research Consortium Initiative. Eur Heart J 2018;**39**(19):1687-1697.

2. Proschan, Michael A., Lan, Gordon K.K., Wittes, Janet, Turk (2006). Statistical Monitoring of Clinical Trials: A Unified Approach. Springer Science+Business Media, LLC, New York, NY
